# Fellow-Workers and the Roll of Honour

**Published:** 1914-12-26

**Authors:** 


### ROUND THE WORLD.

[The Editor Invites Early Information and Contributions Marked "Fellow-Workers."]

### Fellow-Workers and the Roll of Honour.

Captain Thomas Scatchard, R.A.M.C., who was killed early in the war, belonged to a Yorkshire medical family which has for many years been associated with the country round Boston Spa. His preliminary education was obtained at Epsom College, after which he joined the Leeds Medical School and qualified L.M.S.S.A. in 1902. He spent the next three years in gaining clinical experience in various capacities, including those of house physician to the Leeds Infirmary and house surgeon to the Beckett Hospital, Barnsley, and then joined the R.A.M.C. Captain Scatchard was stationed in India for some years, but when war broke out was in charge of the salvarsan department at the Connaught Hospital, Aldershot. Whilst journeying with a number of Staff officers, it appears that a shell burst immediately over the group, instantly killing him, General Finlay, and Colonel Grant Duff.

Lieutenant Arthur Keith Armstrong, R.A.M.C., another victim of the struggle, settled in Monmouth soon after qualifying M.R.C.S., L.R.C.P., from St. Bartholomew's in 1907, and was subsequently appointed to the staff of the local hospital. At the outbreak of war he decided to volunteer for military service, and was accepted by the War Office as one of the additional R.A.M.C. lieutenants then appointed. His probationary course was very short, for on being sent abroad to the Army in France he was killed in action not long after landing.

Lieutenant John Crocket, R.A.M.C., who lost his life at the early age of twenty-eight years, was the nephew of the famous novelist of that name. Educated at Edinburgh, where he took the M.B., Ch.B. only six years ago, he subsequently became house-surgeon to the infirmary and the Royal Hospital for Sick Children there, as well as R.M.O. to the Chalmers' Hospital. Mr. Crocket joined the Army as recently as last year, and, devoting himself to bacteriology, wrote a thesis on "The Noguchi-Luetin Reaction," which gained him the M.D. degree a few months ago.

Captain Frank Forrest, R.A.M.C., whose death has been reported as having occurred in the fighting on the Aisne, studied medicine at Owens College, Manchester, and qualified M.R.C.S., L.R.C.P. ten years ago. Before entering the Army he gained experience as a member of the resident staff of the North Staffordshire Royal Infirmary at Stoke-on-Trent. Being promoted to the rank of captain in 1909, he was holding a post in the Royal Army Medical College when the war broke out.

Lieutenant James Laidlaw Huggan, R.A.M.C., who also fell in one of the skirmishes on the Aisne, was well known outside his professional work as an International Rugby football player. An Edinburgh man, he graduated M.B., Ch.B. three years ago, having done well in all his work as a student; he subsequently held the post of house surgeon at the Edinburgh Royal Infirmary. The late Mr. Huggan displayed the same pluck and prowess in action as had distinguished him on the football field, and his name has appeared in dispatches as having done very brave work in the assistance of the wounded. Just before his death he had been recommended for the Victoria Cross on account of a particularly brilliant feat in which he organised the removal of a number of stricken soldiers from a building that was being heavily shelled.

Captain Richard Crofton George Moore Kinkead, R.A.M.C., news of whose death was received last month, was recalled from Bloemfontein on the outbreak of hostilities, and being given a post with the 10th Hussars went to the Front with them at the beginning of October. A Galway man, he graduated M.B., B.Ch., from Queen's College in 1908, and then took up an appointment as house surgeon to the Coventry and Warwickshire Hospital. The late Captain Kinkead was a keen sportsman, acquitting himself well both on the river and in the hunting-field. As a student he received military training in the South of Ireland Imperial Yeomanry.

Captain Thomas McCann Phillips, R.A.M.C., who was killed in action last month, was an old Queen's College, Belfast, man. He qualified M.B., B.Ch. in 1905, and subsequently held the post of house surgeon at the Royal Victoria Hospital, Belfast. Of late years he had been stationed at Fyzabad, and came home in the early part of the summer to Belfast, where on the outbreak of war he was appointed to the Victoria Barracks. At the end of September he was sent to Aldershot, whence he proceeded to the Front, and was killed during the fierce fighting round Ypres in the early days of the great coast battle. Captain Phillips, whose brother, Dr. Walter Phillips, is on the staff of the Union Medical College at Mukden, was the son of the Rev. J. G. Phillips, of Damascus.

Surgeon Edward Turnbull, R.N., who was lost with H.M.S. *Cressy*, was an Edinburgh and Glasgow man. He qualified M.B. Ch.B.Edin. in 1907, having had a very successful career as a student, in the course of which he had won an important anatomy prize and held the appointment of prosector. Having devoted much of his post-graduate time to the gaining of clinical experience at the Royal Infirmary, Edinburgh, where he was clinical assistant in the surgical wards, house surgeon and resident gynæcologist, the late Mr. Turnbull settled in practice in Berkshire. However, having joined the Royal Naval Volunteer Reserve some two or three years ago, he was called up on mobilisation and joined his ill-fated ship in the first days of August.

Lieutenant William Ormsby Wyndham Ball, R.A.M.C., who was killed the day before his twenty-fifth birthday during the fighting on the Aisne, had already escaped death by a narrow margin on several occasions. During the great retreat his party was constantly under severe fire; on one occasion his horse was killed, and on another a bullet passed through his clothing. The late Mr. W. O. W. Ball was a St. Andrew's, Dublin, man, and graduated M.B., Ch.B. from Dublin University two years ago, after having done very well at Trinity College. He was particularly interested in the formation of the Dublin University Officers' Training Corps, which he joined at the outset. He had only served some eighteen months in the Army before he lost his life.

By an oversight Mr. J. Warrington Haward's name appeared in these notes last week as Mr. J. Warrington Howard.

